# hucMSC-derived exosomes protect ovarian reserve and restore ovarian function in cisplatin treated mice

**DOI:** 10.7555/JBR.36.20220166

**Published:** 2022-11-10

**Authors:** Yue Xiao, Yue Peng, Chi Zhang, Wei Liu, Kehan Wang, Jing Li

**Affiliations:** State Key Laboratory of Reproductive Medicine, Nanjing Medical University, Nanjing, Jiangsu 210029, China

**Keywords:** ovarian function, ovarian reserve, cisplatin, exosomes, apoptosis

## Abstract

Anti-cancer therapy often causes premature ovarian insufficiency and infertility as the ovarian follicle reserve is extremely sensitive to chemotherapy drugs, such as cisplatin. Various fertility preservation methods have been explored for women, especially prepubertal girls undergoing radiotherapy and chemotherapy due to cancer. In recent years, mesenchymal stem cell-derived exosomes (MSC-exos) have been reported to play an important role in tissue repair and the treatment of various diseases. In the current study, we observed that human umbilical cord-derived MSC-exos (hucMSC-exos) after short-term culture improved follicular survival and development while receiving cisplatin treatment. Moreover, intravenous injection of hucMSC-exos improved ovarian function and ameliorated inflammatory environment within the ovary. The underlying mechanism of hucMSC-exos on fertility preservation was associated with the down-regulation of p53-related apoptosis and their anti-inflammatory function. Based on these findings, we propose that hucMSC-exos may be a potential approach to improve fertility in women diagnosed with cancer.

## Introduction

The effects of chemotherapy drugs on female reproduction were reported since the 1970s, which were initially associated with cyclophosphamide (CPM) treatment ^[ 1] ^. There is now ample evidence that cancer treatment can deplete the ovarian reserve, resulting in early menopause ^[ 2] ^. Chemotherapy-induced premature ovarian failure (POF) is a major long-term adverse effect of anti-cancer therapy. Therefore, the development of new approaches to preserve fertility for female patients, especially for those younger patients with a strong desire for childbearing who require chemotherapy drugs, is critical.

*cis*-diamminedichloroplatinum (Ⅱ) (CDDP) is a widely used and effective metal-based chemotherapeutic drug for many types of cancer, including cancers of testicule, ovary, breast, and lung ^[ 3] ^. It is often used in combination with other cytotoxic drugs. Chemotherapeutic drugs, such as CDDP ^[ 4] ^, usually cause double-strand DNA damage, which may induce apoptosis in granulosa cells and oocytes. Females are born with a finite number of primordial follicles, most of which will remain dormant for decades until they are activated into the growing follicular pool ^[ 5] ^. Primordial follicles are very sensitive to the toxicity of chemotherapeutic agents, which may activate the PI3K/AKT signaling pathway that leads to the loss of primordial follicles ^[ 6– 7] ^. Therefore, it is crucial to explore new approaches of protecting the reproductive reserve, while using chemotherapeutic drugs.

In the past decade, mesenchymal stem cells (MSCs) have attracted a great attention due to their reparative function and board therapeutic potential. Human umbilical cord-derived MSCs (hucMSCs) are a type of multipotent stem cell with many advantages, including wide availability, easy accessibility, low immunogenicity and high self-renewal ability ^[ 8] ^. To date, hucMSCs have been studied in clinical therapeutic trials. The stem cell therapy has also emerged as a new treatment for female infertility in recent years. Successful pregnancies have been reported in premature ovarian insufficiency (POI) patients who received hucMSC transplantation ^[ 9] ^. hucMSCs have also been shown to improve the reserve function of perimenopausal ovaries through a paracrine mechanism ^[ 10] ^. However, more research is needed to explore the underlying molecular mechanisms of hucMSCs and how they protect the ovarian reserve.

Many investigators have proved that MSCs secrete products, especially exosomes, to play key roles in tissue repair and healing. Exosomes are extracellular vesicles with a size rage of 30 to 150 nm, which are important in intercellular communication ^[ 11] ^. Similar to their cell source, stem cell-derived exosomes have shown therapeutic effects *via* their reparative and regenerative abilities. It has been shown that MSCs-derived exosomes (MSC-exos) have the same efficacy in tissue repair as MSCs. Recent studies have indicated the potential of hucMSC-derived exosomes (hucMSC-exos) in improving ovarian function in aged mice. However, the role of hucMSC-exos in protecting ovarian reserve and restoring ovarian function after chemotherapy remains unclear. Therefore, we aims to explore whether hucMSC-exos have an ameliorative effect on the damage to ovarian reserve after CDDP chemotherapy. Based on the therapeutic potential of exosomes, we believe that stem cell exosome therapy might represent a new strategy for protecting ovarian reserve during chemotherapy.

## Materials and methods

### Animals

Mice from the Institute of Cancer Research (ICR) were obtained from Nanjing Medical University and housed in the Animal Core Facility of Nanjing Medical University. All mice were maintained under a 12 h light/dark cycle at 22 ℃ with free access to food and water. Female ICR mice at day three were used for ovarian culture. All animal work was approved by the Committee on the Ethics of Animal Experiments at Nanjing Medical University. Superovulation was performed with one intraperitoneal injection of pregnant mare serum gonadotropin (PMSG, 5 IU; NSHF, Ningbo, Zhejiang, China) and followed by another injection of human chorionic gonadotropin (HCG, 5 IU; NSHF) 48 h later.

### *In vitro* fertilization

Donor sperm were first collected from male mice into G-IVF media (Vitrolife, Goteborg, Sweden) and incubated under oil for 1 h at 37 ℃ in 5% CO _2_ for capacitation. Metaphase Ⅱ oocytes were then placed into 250 μL of media with sperm (2 × 10 ^5^/mL to 3 × 10 ^5^/mL) for fertilization. Six hours later, zygotes with clear pronuclei were transferred into fresh G-1 media (Vitrolife) overnight until the two-cell embryonic. The fertilized embryos were cultured in G-1 media for five days to the blastocyst stage.

### The isolation and culture of hucMSCs

Fresh umbilical cords were collected from informed and voluntary mothers at Sir Run Run Hospital of Nanjing Medical University. The experiment protocol was approved by the Ethics of Committee of Nanjing Medical University. Briefly, umbilical cords were dissected into 1 mm ^3^ small pieces and washed with phosphate buffered saline (PBS; Biosharp, Guangzhou, Guangdong, China) containing 100 U/mL penicillin and 100 mg/mL streptomycin (Biofil, Guangzhou, China) for three times. Then tissues were cultured in DMEM/F-12 that contained 10% fetal bovine serum (Gibco, Waltham, MA, USA) and 1% penicillin and streptomycin at 37 ℃ and 5% CO _2_.

### The isolation and characterization of hucMSC-derived exosomes

Briefly, the cultured medium was removed and washed twice with PBS when hucMSC reached 60%–70% confluence. MSCs were then cultured with de-exosome medium for two days. The supernatants were collected and centrifuged at 300 *g* for 10 min at 4 ℃ and centrifuged at 2000 *g* for 10 min to remove dead cells. Then, supernatants were centrifuged at 10 000 *g* for 30 min to remove cell debris and supernatant was then centrifuged at 150 000 *g* for 90 min to acquire exosome pellets. The concentration and particle size were measured by nanoparticle tracking analysis (Particle Metrix, Germany). All procedures were performed at 4 ℃. Exosomes were incubated in 2% paraformaldehyde solution overnight for transmission electron microscopy analysis. Two microliters of the exosome solution were transferred onto a carbon-coated copper grid. Then, the grid was observed using a transmission electron microscope (Tecnai G2 Spirit BioTwin, FEI, Hillsboro, OR, USA).

### *In vitro* mice ovary tissue culture

The ovarian culture-conditioned medium consists of MEM-α (Gibco) supplemented with 0.23 mmol/L pyruvic acid, 50 mg/L streptomycin sulfate, 75 mg/L penicillin G, 0.03 U/mL follicle-stimulating hormone, and 3 mg/mL bovine serum albumin. Cell culture medium of 0.4 mL was added to the bottom of each well, and the postnatal day-three (PD3) ovaries were isolated and cultured in inserts (Millipore, Billerica, MA, USA). Five ovaries were randomly distributed to the control and treated groups. Intact ovaries from PD3 female ICR mice were treated with PBS or CDDP (0.5 μg/mL in PBS; MCE, NJ, USA) combined with or without hucMSC-exos (20 μg/mL) for 24 h, and then transferred to the conditional medium for additional 96-h culture before collection for further analysis.

### The uptake of hucMSC-exos by the cultured newborn ovaries

hucMSC-exos were labeled with PKH67 dye (green) (Sigma, St. Louis, MO, USA) at room temperature for 2 h and ultracentrifugated at 120 000 *g* for 90 min to remove un-conjugated dye. Labeled exosomes were incubated with PD3 ovaries for 24 h. After incubation, ovaries were collected and fixed in 4% formaldehyde for frozen sections. After staining nuclei with Hoechst 33342 (Invitrogen, Carlsbad, CA, USA), slides were observed and calculated by using a confocal laser scanning microscope (LSM, Zeiss, Oberkochen, Germany).

### Follicle counting

For follicle counting *,* ovaries were collected and fixed in 10% formalin overnight. Then, the tissues were cut into serial sections (5 μm thick) and stained by hematoxylin and eosin. To evaluate follicular development, follicles were counted every five sections using the fractionator and nucleator principles ^[ 12] ^. The number of follicles of newborn ovaries was counted from mid-ovary sections. All sections were counted by two individuals.

### Immunohistochemistry and immunofluorescence staining

For immunohistochemistry, sections were deparaffinized and rehydrated, endogenous peroxidase activity was further blocked in 3% hydrogen peroxide in methanol for 15 min. The antigen of ovarian sections were retrieved by high temperature (95 ℃) for 16 min in sodium citrate buffer (pH 6.0). Then the sections were blocked with goat serum for 60 min at room temperature (ZSGB-Bio, Beijing, China), and incubated with primary antibodies at 4 ℃ overnight. The sections were colored with diaminobenzidine (DAB) reagent. For immunofluorescence staining, the sections were incubated with secondary antibody (Alexa Fluor 488 or Alexa Fluor 594; Invitrogen) at 37 ℃ for 60 min followed by the incubation with Hoechst 33342 (Invitrogen).

### Western blotting

Total protein was extracted from ovaries using RIPA buffer (Cat. #P0013B, Beyotime, Shanghai, China) containing 1% protease inhibitor (MCE). Proteins were separated by 10% SDS-PAGE and transferred into polyvinylidene fluoride membranes (Bio-Rad, Hercules, CA, USA). The membranes were incubated with primary antibodies overnight at 4 ℃ after being blocked with Tris-buffered saline with Tween 20 (TBST) containing 5% nonfat dry milk for 60 min. The following primary antibodies were used: Alix (ZENBIO, Chengdu, Sichuan, China), TSG101 (Proteintech, Wuhan, Hubei, China), CD9 (Proteintech), β-tubulin (Abbkine, Wuhan, Hubei, China), γH2AX (abcam, Boston, MA, USA), BAX (CST, Danvers, MA, USA), p53 (CST), Cleaved caspase-3 (CST), Bcl-2 (Abclonal, Wuhan, Hubei, China). Then, the membranes were washed three times with TBST for 10 min and incubated with secondary antibodies for 60 min. The signals were obtained through enhanced chemiluminescence (Thermo Fisher Scientific, Carlsbad, CA, USA) on the Tanon 5200 analysis system (Tanon, Shanghai, China).

### Real-time reverse transcription PCR

Total RNAs were isolated from ovaries using TRIzol reagent (Invitrogen). Total RNAs (500–1000 ng per reaction) were reverse transcribed into cDNAs by FastQuant RT Kit (TIANGEN Biotech, Beijing, China). Real-time reverse transcription PCR (qRT-PCR) was then performed by SYBR Green Mix (Applied Biological Materials, Vancouver, Canada) on an ABI StepOnePlus platform (Thermo Fisher Scientific). All primer sequences are listed in ***Supplementary Table 1*** (available online). The specificity of PCR products was assessed by melting curve analyses.

### Flow cytometric analysis

Cells were fixed in 4% paraformaldehyde with 0.25% Triton X-100 in PBS, and then incubated with primary antibodies at 4 ℃ for 2 h. Primary antibodies purchased from BD Verse (Franklin Lakes, NJ, USA) were used: PE Mouse Anti-Human CD90, APC Mouse Anti-Human CD105, FITC Mouse Anti-Human CD44, FITC Mouse Anti-Human CD34, FITC Mouse Anti-Human 45, and PE Mouse Anti-Human CD11b. After washing with PBST, cells were analyzed by flow cytometry (BD Verse). The data were further analyzed by FlowJo 7.0 software (BD, San Jose, CA, USA).

### Statistics analysis

GraphPad Prism 9.0 and SPSS 20.0 were used to perform the Chi-square test. All data were expressed as mean ± standard deviation and analyzed using unpaired two-tailed Student's *t*-test for two treatment groups and one-way ANOVA followed by Bonferroni's post-hoc multiple comparison correction for multiple treatment groups. *P*< 0.05 was considered statistically significant.

## Results

### The isolation and identification of hucMSC-derived exosomes

**Figure 1:**
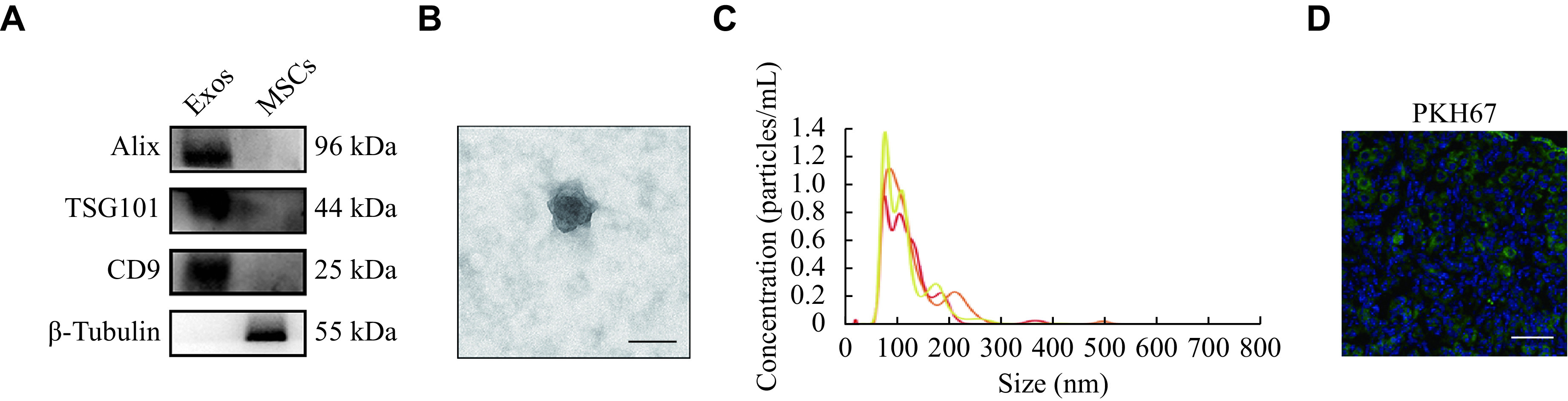
Characterization of hucMSC-derived exosomes.

Flow cytometry was performed to identify surface markers of hucMSCs, and the results showed that CD44, CD90, and CD105 were highly expressed in hucMSCs, while CD34, CD45, and CD11b were negatively expressed ( ***Supplementary Fig. 1 ***, available online). Then the exosome-specific markers Alix, TSG101, and CD9 in hucMSC-exos, isolated from culture supernatant of three to seven passages of hucMSC, were examined by Western blotting, and the results showed that Alix, TSG101, and CD9 were highly expressed in hucMSC-exos ( ***Fig. 1A***). Transmission electron microscopy analysis showed that the obtained hucMSCs-exos were in a round structure with double-layer membranes ( ***Fig. 1B***), and the particles had a typical size of 81 to 109 nm revealed by nanoparticle tracking analysis ( ***Fig. 1C***). These results suggested that exosomes were successfully isolated from hucMSC.

### hucMSC-derived exosomes reduced primordial depletion during cisplatin treatment in newborn mouse ovaries

**Figure 2:**
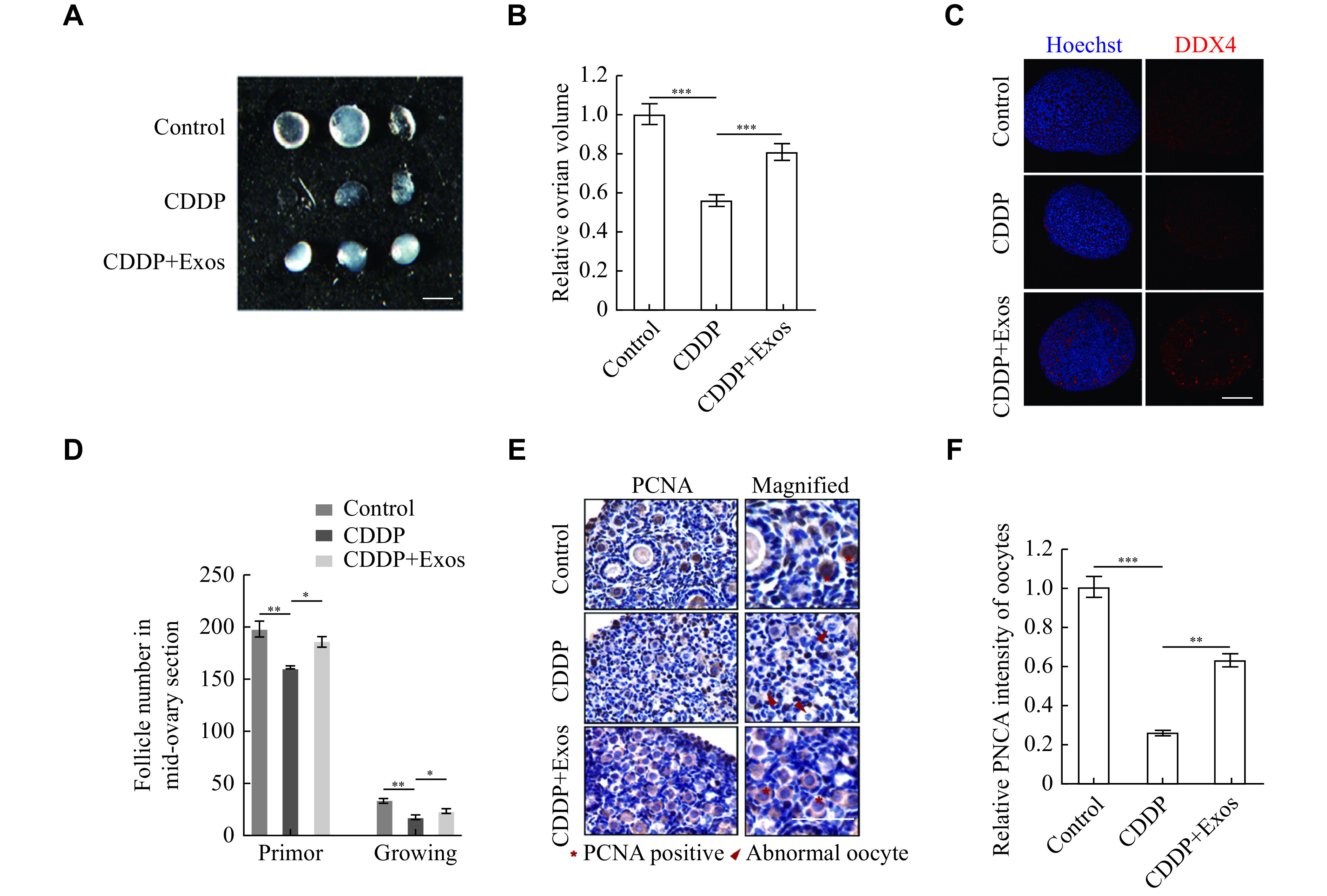
Effects of hucMSC-exos on ovarian reserve after CDDP injury.

To observe the uptake of hucMSC-exos in ovaries *in vitro*, hucMSC-exos were labeled with PKH67 dye for 24 h. As shown in ***Fig. 1D***, PKH67 fluorescence signals were observed to be accumulated in oocytes. The results showed that hucMSC-exos could be uptaken by follicles *in vitro*. Several studies have shown that chemotherapy can destroy immature follicles like primordial follicles as well as secondary ones ^[ 13] ^. Newborn ovaries at PD3, which contain the mainly amount of ovarian reserve, were used to study the effects of hucMSC-exos on ovarian reserve protection during CDDP treatment. The results showed that CDDP treatment significantly reduced the volume of ovaries, compared with the control group, but hucMSC-exos treatment significantly attenuated CDDP-induced reduction of ovarian volume ( ***Fig. 2A*** and ***2B***).

To assess follicular development, immunofluorescent staining of DEAD-box polypeptide 4 (DDX4) was used to localize oocytes. The results showed that the DDX4 staining could be clearly observed in primordial follicle cytoplasm ( ***Fig. 2C***). The number of follicles was counted from mid-ovary sections after treatments. We found a significant decrease in the number of both primordial and growing follicles in the ovaries of the CDDP group, compared with that of the control group, but hucMSC-exos treatment reduced CDDP-induced loss of primordial and growing follicles in ovaries ( ***Fig. 2D***). Immunohistochemistry staining of the proliferating cell nuclear antigen (PCNA) in ovaries showed a decreased PCNA expression and more abnormal oocytes in the CDDP-treated group, compared with the control group as well as the increased PCNA expression and less abnormal oocytes in the CDDP + hucMSC-exos group, compared with the CDDP group ( ***Fig. 2E*** and ***2F***). These results indicated that CDDP treatment directly destroyed ovarian reserve and hucMSC-derived exosomes could protect primordial follicles to a certain extent.

### hucMSC-exos treatment inhibited follicle apoptosis and DNA breakage in oocytes *in vitro*

**Figure 3:**
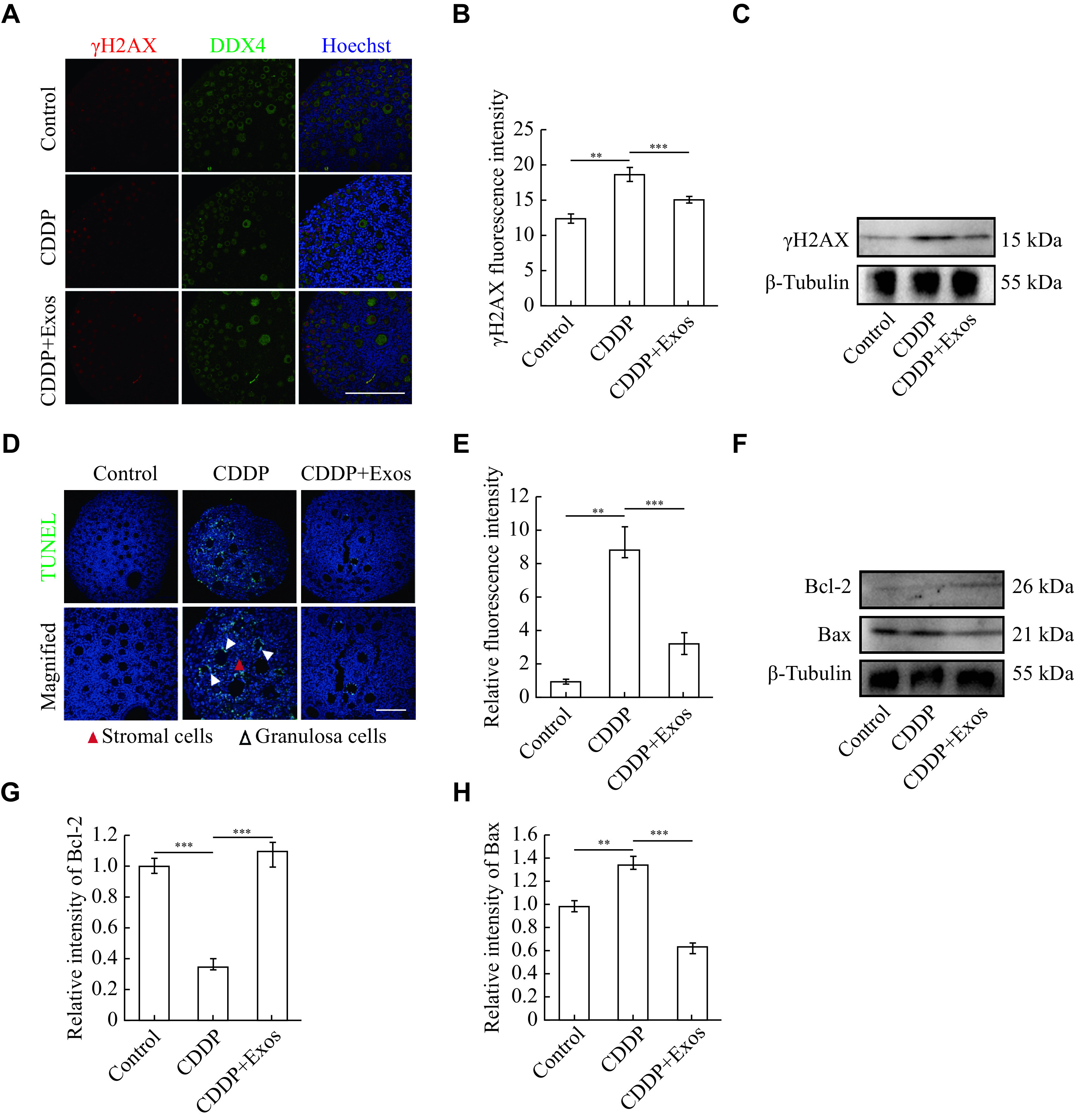
hucMSC-exos ameliorated CDDP-induced follicular apoptosis and DNA breakage in primordial oocytes.

Previous studies have reported that CDDP treatment leads to follicle apoptosis and DNA damage ^[ 14– 15] ^. Immunofluorescence staining of phosphorylated histone H2AX (γH2AX), a marker of DNA damage, revealed a significant increase in the number of double-strand breaks (DSBs) in CDDP treated ovaries, which was reversed in ovaries of the CDDP + hucMSC-exos group ( ***Fig. 3A*** and ***3B***). Moreover, γH2AX expression detected by Western blotting was lower in the CDDP + hucMSC-exos group than in the CDDP group ( ***Fig. 3C***). These results suggested that hucMSC-exos could repair ovarian DNA double-strand damage.

The apoptosis level in ovaries was detected by TdT-mediated dUTP-biotin nick end-labeling (TUNEL) assay, and the results showed a higher level of apoptosis of granulosa cells and stromal cells in CDDP-treated ovaries, compared with the control group, but the apoptosis level was returned to that similar to the control group after hucMSC-exos treatment following CDDP challenge ( ***Fig. 3D*** and ***3E***). The gene *Bcl2* and *Bax* play important roles in apoptosis ^[ 16] ^. The effects of CDDP and hucMSC-exos on Bcl-2/Bax apoptotic pathway in ovaries were analyzed by Western blotting. The results showed that Bcl-2/Bax ratio was significantly down-regulated in the CDDP group, compared with the control group, and up-regulated in the CDDP + hucMSC-exos group, compared with the CDDP group ( ***Fig. 3F–3H***). These results suggested that hucMSC-exos could preserve follicles by inhibiting oocyte DNA breakage and apoptosis in ovaries.

### hucMSC-exos improved ovarian follicle function and restored fertility in CDDP-treated mice *in vivo*

**Figure 4:**
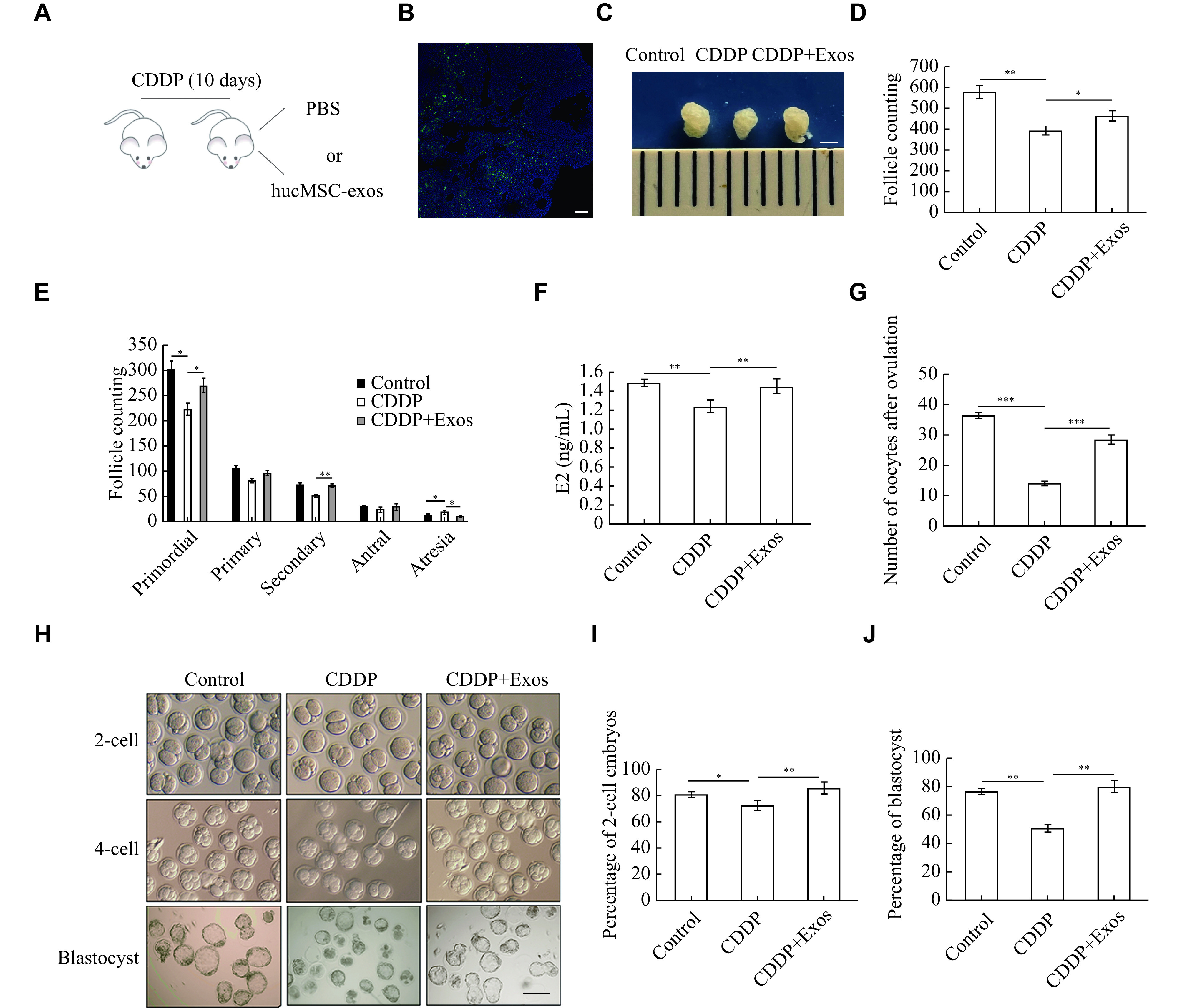
hucMSC-exos improved follicular development rescue of ovulation of mature eggs and embryonic development.

A diagram scheme for *in vivo* experiment was shown in ***Fig. 4A***. Briefly, three-week old female mice received intraperitoneal injection of CDDP for 10 days and intravenous injection of PBS or hucMSC-exos for two more weeks. hucMSC-exos were labeled with PKH67 dye for tracing, and a strong green fluorescent protein (GFP) signal was detectable in the ovarian medulla and surrounding follicles ( ***Fig. 4B***). Two weeks after PBS/exosomes injection, the ovarian volume of the CDDP + hucMSC-exos group was greater than that of the CDDP group ( ***Fig. 4C***). Total follicle counting showed a significant loss of follicle in the CDDP group and a retention of follicles in the CDDP + hucMSC-exos group ( ***Fig. 4D***). Follicles counting results revealed that, compared with controlled ovaries, the CDDP-treated ones revealed extreme damage in terms of follicular development, including a decreased follicle count at developmental stages and an increase in atretic follicles ( ***Fig. 4E***). In contrast, ovaries received hucMSC-exos treatment following CDDP challenge showed an obvious improvement in the number of growing follicles at different developmental stages compared with the CDDP group ( ***Fig. 4E***).

The concentration of serum estradiol (E2) was measured using an enzyme-linked immunosorbent assay. Compared with the control group, CDDP treatment induced a significant down regulation of E2 in the serum, which was attenuated by hucMSC-exos treatment ( ***Fig. 4F***). We then examined the function of hucMSC-exos for ovulation and mature egg production. After superovulation, an increased number of ovulated oocytes were harvested from the mice of the CDDP + hucMSC-exos group, compared with the CDDP group ( ***Fig. 4G***). These results suggested that the *in vivo* hucMSC-exos treatment improved oocyte retrieval.

Then, we evaluated the *in vitro* fertilization (IVF) ability of oocytes from each group. After IVF with the donor sperm, the rates of two-cell embryos to blastocysts in the CDDP group were reduced, but they were rescued by hucMSC-exos transplantation ( ***Fig. 4H***– ***4J***). These results indicated that hucMSC-exos played a protection role in cisplatin-induced follicle damage and restored follicular development *in vivo* to enhance fertility.

### hucMSC-exos protected granulosa cell from CDDP damage

**Figure 5:**
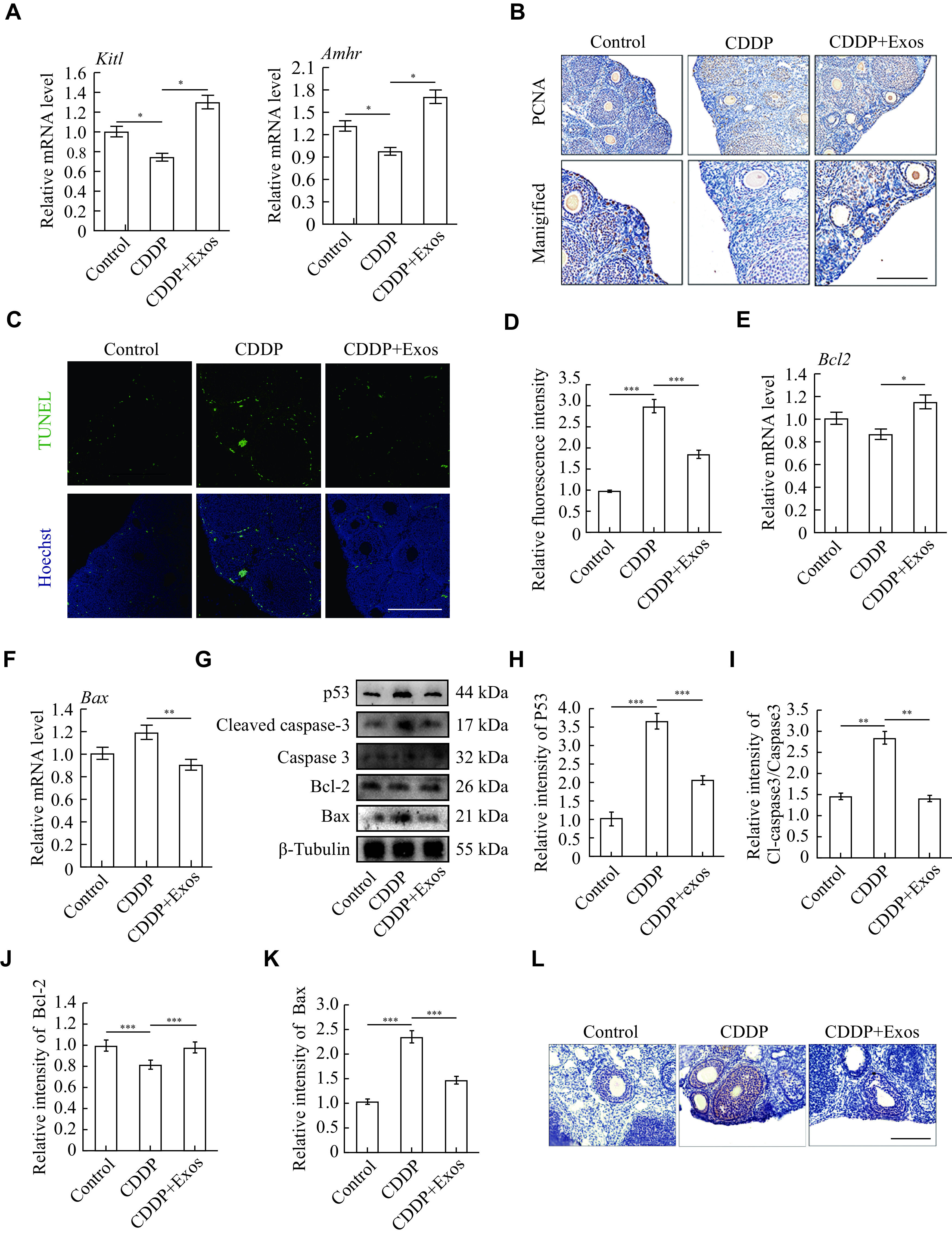
The improvement of ovarian granulosa apoptosis in CDDP-treated ovaries.

In the neonatal mouse ovary culture model, we observed an increased level of apoptosis in early follicular granulosa cells after CDDP treatment. Therefore, we explored whether hucMSC-exos could improve follicle development by restoring granulosa cell function. The expression levels of granulosa cell-associated genes ( *i.e.*, *Kitl* and *Amhr*) in ovaries were detected by qRT-PCR. The results showed that the expression levels of *Kitl* and *Amhr* were significantly upregulated in ovaries of the CDDP + hucMSC-exos group, compared with the CDDP group ( ***Fig. 5A***). Immunostaining of PCNA showed that more positive signals were detected in granulosa cells in the CDDP + hucMSC-exos group, compared with the CDDP group ( ***Fig. 5B***). These results indicated that hucMSC-exos promoted granulosa cell proliferation and improved granulosa functions.

Results of the TUNEL assay further demonstrated that apoptosis in the ovaries was increased in the CDDP group, compared with the control group, and decreased in CDDP + hucMSC-exos group, compared with the CDDP group ( ***Fig. 5C*** and ***5D***). The apoptosis level in early granulosa cells was increased in the CDDP group. To demonstrate the protective role of hucMSC-exos against cells damage in the ovaries, we dissociated primary ovarian granulosa cells from ovaries and cultured them *in vitro*. The mRNA expression levels of anti-apoptosis gene *Bcl2* and pro-apoptosis gene *Bax* in granulosa cells were detected by qRT-PCR. The results showed that *Bcl2* expression was significantly increased in the CDDP + hucMSC-exos group, compared with the CDDP group, while *Bax* expression was significantly reduced in the CDDP + hucMSC-exos group, compared with the CDDP group ( ***Fig. 5E*** and ***5F***). Western blotting was performed to detect the expression of apoptosis-related proteins in granulosa cells. Compared with the CDDP group, the expression levels of p53 and cleaved caspase-3 were down-regulated, and the Bcl-2 level was also up-regulated in the CDDP + hucMSC-exos group ( ***Fig. 5G–5K***). These results indicated a decrease in the level of apoptosis in the ovary after hucMSC-exos treatment. Similarly, immunohistochemistry staining of cleaved caspase-3 showed that hucMSC-exos treatment reduced cell apoptosis in the ovaries induced by CDDP ( ***Fig. 5L***). These results consistently showed that hucMSC-exos had a protective effect against CDDP-induced granulosa damage.

### hucMSC-exos improved inflammatory environment in the ovaries

**Figure 6:**
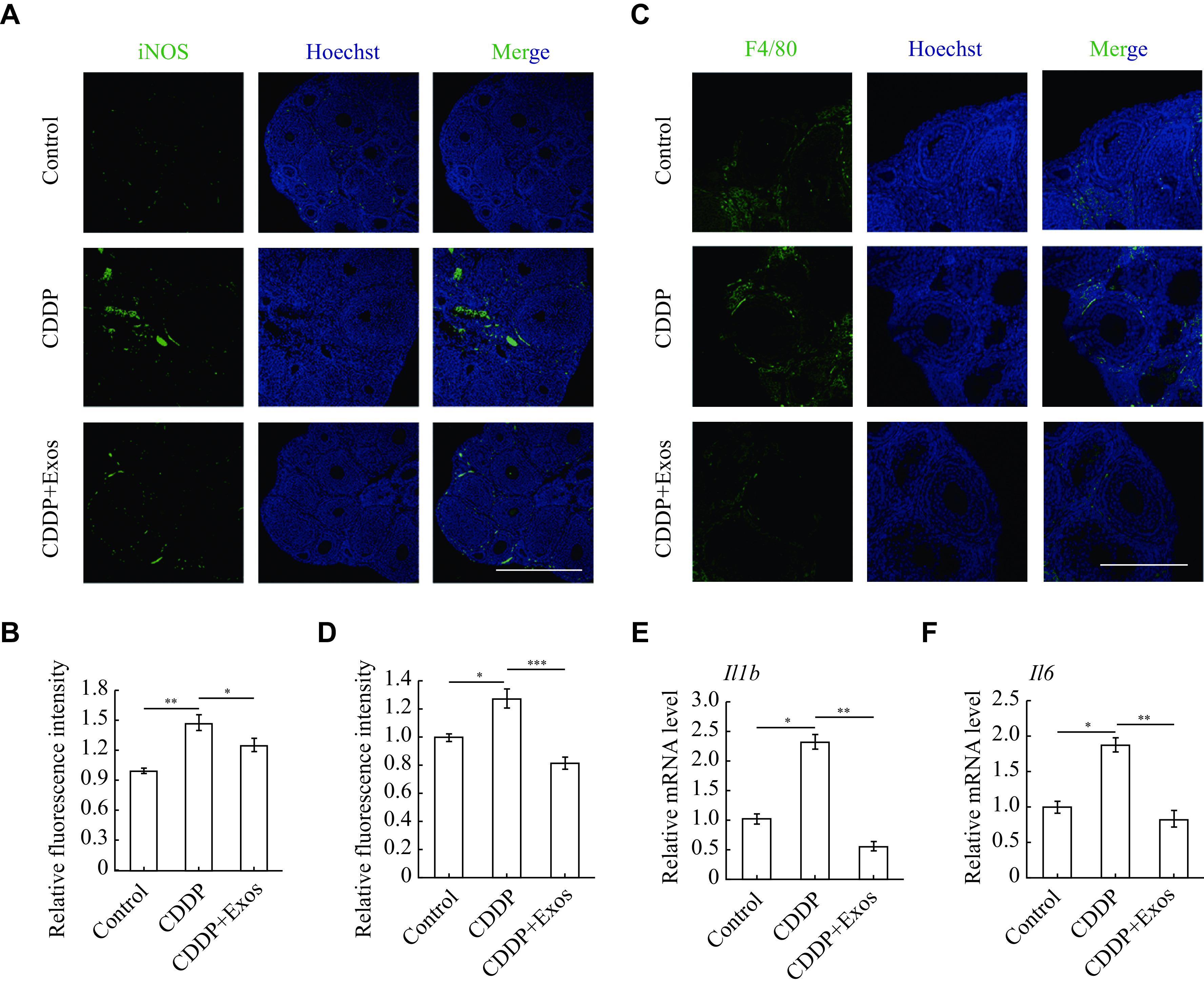
hucMSC-exos changed ovarian inflammatory environment in CDDP-treated ovaries.

CDDP has been reported to induce pro-inflammatory signaling, which is associated with persisting markers of inflammation ^[ 17] ^. We next evaluated whether there were beneficial effects of hucMSC-exos on CDDP-treated ovaries. The distribution of M1-like macrophages was detected by immunofluorescence staining of inducible nitric oxide synthase (iNOS). The results showed the decreased positive signals for M1-like macrophages in the hucMSC-exos group, compared with the CDDP group ( ***Fig. 6A*** and ***6B***). The results of immunofluorescence staining of F4/80 showed that macrophage infiltration in the ovaries was increased in the CDDP group, compared with the control group and decreased in the CDDP + hucMSC-exos group, compared with the CDDP group ( ***Fig. 6C*** and ***6D***). The results of qRT-PCR showed that hucMSC-exos treatment could significantly suppress CDDP-induced increase in the expression of pro-inflammatory genes, including *Il1b* and *Il6* ( ***Fig. 6E*** and ***6F***). Taken together, the results suggested that hucMSC-exos treatment could alter the inflammatory environment in CDDP-treated ovaries.

## Discussion

Women of childbearing age often experience POF and infertility due to the damaging effect of chemotherapy drugs, which are regular side effects of anti-cancer therapies. How to prevent POF and protect follicle pools among this population with fertility aspirations have received increasing attention. At present, the clinical application to prevent ovarian function damage by chemotherapy is not very extensive. The main current approaches to fertility preservation after chemotherapy include administration of sphingosine-1-phosphate ^[ 18] ^, melatonin ^[ 19] ^, and AS101 ^[ 20– 21] ^. In the current study, we proposed and proved that hucMSC-exos had a protective effect on follicular protection after chemotherapy ( *e.g.*, cisplatin) treatment. Additionally, in hucMSC-Exos-treated ovaries, we found follicle developmental restoration. hucMSC-exos significantly increased the number of eggs that ovulated from superovulated ovaries, as well as blastocyst formation. These findings are consistent with our previous reports about the fertility restoration in aged ovaries after hucMSC-exos administration ^[ 10] ^. Our findings suggest that hucMSC-exos may be another candidate to preserve ovarian reserve and improve ovarian function after CDDP chemotherapy.

As the most common chemotherapy drug, cisplatin is still the first-line chemotherapeutic drug for the treatment of several cancers, including breast cancer, and widely used in clinical treatment of hematologic and solid tumors in children and women of childbearing age. However, it is still unclear how is the mechanism of CDDP exposure in ovarian failure. There is a growing evidence that exposure to CDDP leads to follicular excessive consumption and loss ^[ 22] ^. The proposed mechanism of CDDP to damage follicles may be through the increase of DNA damage and the activation of apoptosis related genes, like *P53* family, especially in immature follicles ^[ 23] ^. In the current study, the significant increase in γH2AX signals in CDDP-cultured ovaries indicates that cisplatin induces DSBs in oocytes, leading to ovarian reserve damage. Notably, a decrease in the number of DSB positive-labeled oocytes was observed after hucMSC-exos treatment. These findings suggest that hucMSC-exos treatment may repair DNA DSBs in oocytes. The observation likely explains why more primordial follicles preservation detected in newborn ovaries.

Studies have reported a pro-apoptosis function induced by CDDP ^[ 24] ^. p53 is a tumor suppressor protein that regulates cell growth by modulating its target genes *P27*, *Bcl2*, *Bax*, and *Puma* (the gene encoding p53 up-regulated modulator of apoptosis). In the current study, we observed that the apoptosis of granulosa cells and DSBs in oocytes increased after CDDP treatment, which is consistent with the effects of CDDP on the ovaries in a rat model ^[ 25] ^. Investigators have pointed out that the activation of p53 can induce apoptosis and cell migration ^[ 26] ^. Here, we found that hucMSC-exos up-regulated Bcl-2 protein expression and down-regulated cleaved caspase-3 protein expression to protect granulosa cells from CDDP injury. Moreover, hucMSC-exos reduced the apoptotic sign in ovarian follicles, which suggests hucMSC-exos may improve ovarian function. In our current study, we found that the expression of p53 was decreased in granulosa cell cultured by CDDP. After hucMSC-exos treatment, we were surprised to find that embryonic development after IVF was improved. It also indirectly demonstrates that hucMSC-exos improve follicular development by the restoration of granulosa function.

With the developments in stem cell therapy, many investigators focus on the use of adipose-derived MSCs or bone marrow-derived MSCs in the regeneration and functional recovery *via* experimental animal models ^[ 27– 28] ^. Compared with other sources of stem cells, hucMSCs are the first choice for cell therapy due to their non-invasive isolation, low immunogenicity and the ability of fast self-renewal ^[ 29] ^. However, hucMSCs have relative limitations in the quantification of bioactive substances and the logistics for delivery in clinical therapies. Several investigators suggested that the culture medium of MSCs had therapeutic effects in POI murine models ^[ 30] ^. Compared with MSCs, exosome, a member of extracellular vesicles may be a better choice in the clinic, because of their low immunogenicity, high clinical safety and stable bioactivity ^[ 31] ^. Therefore, the application of MSC-exos is more effective and safer than the MSC therapy.

The successful maturation of oocytes is associated with systemic hormone regulation, granulosa cell function, and ovarian microenvironment. Similarly, the homeostasis of the perifollicular environment depends on the maintenance of multiple cells, including immune cells and stromal cells. CDDP induces a myriad of inflammatory cytokines and chemokines, including translocation factors, which lead to the production of tumor necrosis factor alpha (TNF-α), a pro-inflammatory cytokine that is actively involved in CDDP-induced inflammation. Therefore, we examined the expression of inflammatory factors in the ovaries after CDDP treatment. The expression of major proinflammatory cytokines IL-1β and IL-6 increased in CDDP-treated ovaries. The increased number of macrophages found in the ovary after CDDP treatment indicates that CDDP is correlated with a proinflammatory environment in ovaries. To be noted, hucMSC-exos improved ovarian follicular development, meanwhile the anti-inflammatory effects of hucMSC-exos were required for the amelioration of ovarian microenvironment and improved ovarian function in the CDDP-treated mice.

The molecular mechanism, involved in hucMSC-exos repair of the ovarian function, needs to be further explored. The active components in hucMSC-exos may regulate ovarian microenvironment. We will focus on what components of hucMSC-exos are involved in the recovery of ovarian function in future studies.

## Ackonwledgements

None.
